# Prognostic associations of PD-L1 expression and BAP1 loss in pleural mesothelioma: a retrospective single-center cohort from Colombia

**DOI:** 10.3389/fonc.2026.1920798

**Published:** 2026-10-14

**Authors:** Ricardo Brugés, Carlos Carvajal, Angela Luna-Meza, Marcela Núñez, Alejandra Navarrete, Rafael Parra-Medina

**Affiliations:** 1 Departamento de Oncología, Instituto Nacional de Cancerología, Bogotá, Colombia; 2 Departamento de Cirugía de Tórax, Instituto Nacional de Cancerología y Fundación CTIC, Bogotá, Colombia; 3 Departamento de Oncología, Clínica Universitaria Colombia, Fundación Universitaria Sanitas, Bogotá, Colombia; 4 Departamento de Estadística, Instituto Nacional de Cancerología, Bogotá, Colombia; 5 Departamento de Patología Oncologica, Instituto Nacional de Cancerología, Bogotá, Colombia; 6 Instituto de Investigación, Universidad FUCS., Bogotá, Colombia

**Keywords:** asbestos, chemotherapy, Colombia, Latin America, low- and middle-income countries, mesothelioma, survival

## Abstract

**Background:**

Evidence on the prognostic associations of programmed death-ligand 1 (PD-L1) and BRCA1-associated protein 1 (BAP1) in pleural mesothelioma (PM) remains inconsistent, and joint data from Latin American cohorts are limited. We evaluated these biomarkers in a Colombian cohort.

**Methods:**

This retrospective single-center cohort included adults with histologically confirmed PM treated at the Instituto Nacional de Cancerología, Bogotá, from January 2011 through December 2023. PD-L1 was quantified using tumor proportion score (TPS), with ≥1% as the primary threshold; BAP1 was classified as retained or lost by nuclear immunoreactivity. Overall survival (OS) was measured from pathologic diagnosis to death. Event-free survival (EFS) was measured from diagnosis to first documented progression or death; patients without an event were censored at last contact. Kaplan–Meier methods and separate multivariable Cox models were used.

**Results:**

Sixty-seven patients were included; 60/67 (89.6%) had locally advanced or metastatic disease. PD-L1 TPS ≥1% was present in 26/67 (38.8%), and BAP1 loss in 47/61 evaluable tumors (77.0%). All 67 patients died. Median OS was 9.20 months (95% CI, 6.11–14.13), and median EFS was 6.51 months (95% CI, 5.49–9.13). PD-L1 TPS ≥1% was not clearly associated with OS (adjusted HR, 1.03; 95% CI, 0.57–1.86) or EFS (adjusted HR, 1.11; 95% CI, 0.62–2.00). BAP1 loss also showed no clear adjusted association with OS (HR, 1.57; 95% CI, 0.78–3.16) or EFS (HR, 1.65; 95% CI, 0.82–3.33).

**Conclusions:**

Neither PD-L1 TPS ≥1% nor BAP1 loss showed a clear adjusted association with survival. These jointly measured estimates extend the evidence from an underrepresented Latin American population but require external validation; the exploratory ≥5% and ≥10% analyses were based on only 14 and 7 patients, respectively.

## Introduction

Pleural mesothelioma (PM) is an uncommon asbestos-related cancer with poor survival. A contemporary analysis of 41,074 US cases reported a median overall survival (OS) of 10.3 months, and histologic subtype and epithelioid grade remain clinically relevant prognostic features (1–3).

BRCA1-associated protein 1 (BAP1) loss is a highly specific immunohistochemical marker that helps distinguish PM from reactive mesothelial proliferations (4, 5). Its association with survival has varied across clinicopathologic cohorts (6–9). Programmed death-ligand 1 (PD-L1) expression has also been associated with adverse prognosis in some studies, including a meta-analysis, although assay methods, thresholds, histologic composition, and adjustment strategies differ substantially (10–12). Prognostic association should be distinguished from treatment prediction: randomized evidence supports immune checkpoint inhibition in unresectable PM, but PD-L1 has not been established as a stand-alone marker for selecting treatment (13, 14).

Latin American cohorts have described clinical outcomes in PM and, in Mexico, the association between PD-L1 and prognosis (15–17). Evidence from the region is therefore not absent, but joint evaluation of PD-L1 and BAP1 remains limited. Assessing both markers in the same cohort can test whether an immune-evasion marker and a tumor-suppressor marker provide complementary prognostic information while also documenting their local prevalence.

We aimed to estimate the separate associations of PD-L1 TPS ≥1% and BAP1 loss with OS and diagnosis-based event-free survival (EFS) in a retrospective Colombian cohort. Higher PD-L1 thresholds and combined biomarker groups were evaluated as exploratory analyses.

## Methods

### Study design and setting

We conducted a retrospective single-center cohort study at the Instituto Nacional de Cancerología (INC), a national tertiary oncology referral hospital in Bogotá, Colombia. The cohort included patients seen between January 1, 2011, and December 31, 2023.

### Patient selection

Eligible patients were adults aged 18 years or older with histologically confirmed PM who attended the thoracic surgery or medical oncology services during the study period and had at least one biomarker result available. Patients remained eligible for the overall cohort if BAP1 was not evaluable or had not been performed. The analytic dataset contained the 67 included patients but did not include records of pre-inclusion screening.

The PD-L1 analysis population included all 67 patients. BAP1 and combined-biomarker analyses were restricted to the 61 patients with evaluable BAP1 results. Complete-case adjusted models included 64 patients for PD-L1 analyses and 58 for BAP1 analyses because stage was missing in three patients. Available analysis populations are reported in **Figure 1**; pre-inclusion screening counts were unavailable.

**Figure 1:**
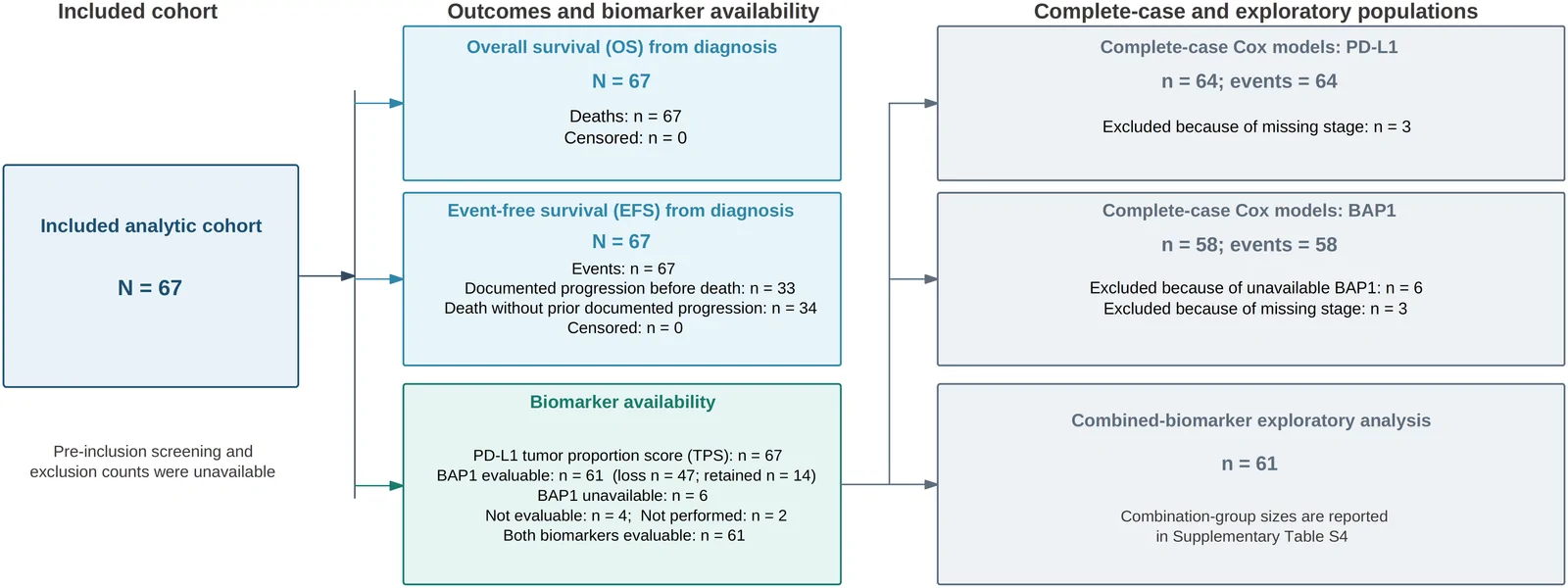
Study cohort and analysis populations. Flow diagram of the included cohort, outcome populations, biomarker availability, and complete-case Cox models. Pre-inclusion screening and exclusion counts were unavailable. OS, overall survival; EFS, event-free survival; PD-L1, programmed death-ligand 1; TPS, tumor proportion score; BAP1, BRCA1-associated protein 1.

### Specimen processing and immunohistochemistry

One pathologist reviewed available formalin-fixed, paraffin-embedded primary or metastatic tumor material to confirm histologic subtype, tumor content, and suitability for additional staining. Epithelioid grade was reassigned according to the fifth edition of the WHO classification (18). Formal outcome blinding was not documented in the retrospective source records.

Four-micrometer sections were stained on a Ventana BenchMark ULTRA platform with ready-to-use anti–PD-L1 SP142 antibody and anti-BAP1 mouse monoclonal antibody (clone C-4, sc-28383). PD-L1 TPS was defined as the percentage of viable tumor cells with partial or complete membranous staining of any intensity; staining across viable tumor areas was included when heterogeneous. The primary threshold was TPS ≥1%. BAP1 loss was defined as complete absence of tumor-cell nuclear staining in the presence of retained nuclear staining in non-neoplastic internal controls. Specimens with insufficient viable tumor or uninterpretable control staining were classified as not evaluable; assays not undertaken were classified as not performed. Placental and tonsil tissues served as external positive controls.

### Measurements and exposures

Age, sex, comorbidities, Eastern Cooperative Oncology Group (ECOG) performance status, tumor laterality, histology, epithelioid grade, American Joint Committee on Cancer (AJCC) eighth-edition stage, treatment, and laboratory values closest to diagnosis were abstracted from the clinical record. Asbestos and tobacco exposure were based on exposure history documented in the medical record. The category “none documented” indicated that neither exposure was recorded and was not interpreted as confirmed absence.

For descriptive reporting, stage was grouped as early (IA/IB), locally advanced (IIIA/IIIB), metastatic (IV), or missing. Anticancer treatment pathways were defined as mutually exclusive: oncologic surgery with or without systemic therapy or radiotherapy; systemic therapy without oncologic surgery; chemotherapy prescribed but not received; and no systemic therapy prescribed. Palliative care was reported separately as a supportive-care service and was not treated as absence of care.

PD-L1 TPS ≥1% was the primary biomarker threshold. TPS ≥5% and TPS ≥10% were examined only as exploratory thresholds. BAP1 was analyzed as loss versus retained among evaluable tumors. Combined PD-L1/BAP1 groups were exploratory because of small cell sizes.

### Outcomes

The primary outcome was OS, defined from the date of pathologic diagnosis to death from any cause. Patients alive at data cutoff would have been censored at last documented contact. The secondary outcome was EFS, defined from the date of pathologic diagnosis to the first documented radiologic or clinical progression or death from any cause. Patients with neither event would have been censored at last documented contact. All 67 included patients entered both outcome analyses. Vital status and dates of death were cross-checked against the national ADRES-BDUA registry.

### Data handling

Data were abstracted from the electronic medical record into a secure REDCap database and checked before analysis. Missing values were reported by variable. Survival outcomes and PD-L1 TPS were available for all 67 patients. BAP1 was evaluable in 61 patients, and stage was available in 64. Primary Cox models used complete cases; no multiple imputation was performed, and non-evaluable BAP1 results were not imputed or combined with retained or loss categories.

### Statistical analysis

Analyses were performed in R version 4.6.1 (R Foundation for Statistical Computing, Vienna, Austria) (19). Categorical variables were summarized as n (%), normally distributed continuous variables as mean (SD), and skewed variables as median (interquartile range). Two-sided p values were reported, with p < 0.05 used as the conventional statistical threshold.

OS and EFS were estimated using the Kaplan–Meier method, with unadjusted groups compared using two-sided log-rank tests. Median follow-up was assessed with the reverse Kaplan–Meier method. Cox proportional hazards models estimated hazard ratios (HRs) and 95% confidence intervals (CIs). To limit overfitting and avoid conditioning on post-diagnosis treatment, separate multivariable models were fitted for PD-L1 and BAP1. Each model included age per 10-year increase, sex, ECOG 2–3 versus 0–1, non-epithelioid versus epithelioid histology, and stage IV versus I–III. Stage was dichotomized as stage IV versus stages I–III because only four patients had early-stage disease, which would have provided an unstable reference group in a more granular model. These covariates were selected *a priori* for their established clinical relevance and consistency across biomarker models. Treatment was excluded to avoid conditioning on a post-diagnosis exposure and because of confounding by indication. Platelet count was not prespecified in the adjustment set and was omitted from the primary models. Proportional hazards assumptions were evaluated using Schoenfeld residuals.

PD-L1 TPS ≥5% and ≥10%, histology-stratified curves, and the four combined PD-L1/BAP1 groups were exploratory. Because only 14 and 7 patients met the higher TPS thresholds and combined groups were small, these analyses were limited to unadjusted estimates and placed in the Supplement. No adjusted four-group combined-biomarker model was fitted.

### Ethics

The study was conducted in accordance with the Declaration of Helsinki, the Council for International Organizations of Medical Sciences guidelines, and Colombian Resolution 8430 of 1993. The Research and Ethics Committee of the Instituto Nacional de Cancerología, Bogotá, Colombia, approved the protocol (version 02, November 30, 2023; approval/reference no. CE-01080-22) and waived informed consent because the study used retrospective de-identified data and archived specimens.

## Results

### Baseline characteristics

The analytic cohort included 67 patients with histologically confirmed PM. Pre-inclusion screening and exclusion data were unavailable; the resulting analysis populations are shown in **Figure 1**. Mean age was 64.9 years (SD, 9.5), 52/67 (77.6%) patients were male, and 50/67 (74.6%) had ECOG performance status 0–1. Epithelioid histology was present in 57/67 (85.1%). Stage was locally advanced in 22/67 (32.8%) and metastatic in 38/67 (56.7%), for a combined 60/67 (89.6%); stage was missing in three patients. Among 56 evaluable epithelioid tumors, 34 (60.7%) were high grade. Asbestos exposure, alone or with tobacco exposure, was documented in 32/67 (47.8%) (**Table 1**).

**Table 1:** Baseline demographic, clinical, pathologic, and biomarker characteristics.

Characteristic	Category/statistic	Value, n (%)
Age, years	Mean (SD)	64.9 (9.5)
Age ≥65 years	Yes	38 (56.7)
Sex	Female	15 (22.4)
	Male	52 (77.6)
Any recorded comorbidity	Yes	40 (59.7)
	Diabetes	11 (16.4)
	Hypertension	10 (14.9)
Exposure history from clinical record	Asbestos only	10 (14.9)
	Tobacco only	15 (22.4)
	Asbestos and tobacco	22 (32.8)
	Neither exposure documented	20 (29.9)
Body mass index, kg/m²	Mean (SD)	22.9 (4.0)
	Missing data	4 (6.0)
ECOG performance status	0	8 (11.9)
	1	42 (62.7)
	2	14 (20.9)
	3	3 (4.5)
Tumor laterality	Right	33 (49.3)
	Left	34 (50.7)
AJCC 8th-edition stage	IA	2 (3.0)
	IB	2 (3.0)
	IIIA	6 (9.0)
	IIIB	16 (23.9)
	IV	38 (56.7)
	Missing data	3 (4.5)
Grouped stage	Early (IA/IB)	4 (6.0)
	Locally advanced (IIIA/IIIB)	22 (32.8)
	Metastatic (IV)	38 (56.7)
	Missing data	3 (4.5)
Histologic subtype	Epithelioid	57 (85.1)
	Biphasic	5 (7.5)
	Sarcomatoid	5 (7.5)
Epithelioid grade	High	34 (60.7)
	Low	22 (39.3)
	Missing data	1 (1.8)
PD-L1 TPS	<1%	41 (61.2)
	≥1%	26 (38.8)
	≥5% (exploratory)	14 (20.9)
	≥10% (exploratory)	7 (10.4)
BAP1 status	Retained	14 (20.9)
	Loss	47 (70.1)
	Missing data	6 (9.0)

Percentages were calculated using the applicable denominator for each variable. Epithelioid grade was assessed only among epithelioid tumors (n=57); grading data were missing for one tumor, and non-epithelioid tumors were not included in this denominator. BAP1 data were missing for six patients (four tumors were not evaluable and testing was not performed in two). “Neither exposure documented” does not establish absence of exposure. AJCC, American Joint Committee on Cancer; ECOG, Eastern Cooperative Oncology Group; PD-L1, programmed death-ligand 1; TPS, tumor proportion score; BAP1, BRCA1-associated protein 1; SD, standard deviation.

### Biomarker status

PD-L1 TPS ≥1% was present in 26/67 patients (38.8%). BAP1 was evaluable in 61 patients: 47/61 (77.0%) showed loss and 14/61 (23.0%) retained expression; four assays were not evaluable and two were not performed. Histology-specific denominators are reported in **Table 2**. Both biomarkers were evaluable in 61 patients. The combined groups were PD-L1 TPS <1%/BAP1 loss (n = 30), PD-L1 TPS <1%/BAP1 retained (n = 7), PD-L1 TPS ≥1%/BAP1 loss (n = 17), and PD-L1 TPS ≥1%/BAP1 retained (n = 7). Baseline comparisons by biomarker status are provided in **Supplementary Tables S1, S2**.

**Table 2:** PD-L1 TPS and BAP1 status by histologic subtype.

Histology	Subtype n	PD-L1 TPS <1%, n/N (%)	PD-L1 TPS ≥1%, n/N (%)	BAP1 retained, n/N (%)	BAP1 loss, n/N (%)	BAP1 unavailable, n/N (%)
Epithelioid	57	36/57 (63.2)	21/57 (36.8)	13/52 (25.0)	39/52 (75.0)	5/57 (8.8)
Biphasic	5	3/5 (60.0)	2/5 (40.0)	0/4 (0.0)	4/4 (100.0)	1/5 (20.0)
Sarcomatoid	5	2/5 (40.0)	3/5 (60.0)	1/5 (20.0)	4/5 (80.0)	0/5 (0.0)
Total	67	41/67 (61.2)	26/67 (38.8)	14/61 (23.0)	47/61 (77.0)	6/67 (9.0)

PD-L1 TPS was available for all 67 patients. BAP1 percentages for retained and loss use evaluable tumors within each histologic subtype. BAP1 unavailable combines four not-evaluable and two not-performed assays. TPS, tumor proportion score.

### Treatments received

Anticancer treatment pathways were mutually exclusive: five patients (7.5%) underwent oncologic surgery with or without systemic therapy or radiotherapy, 36 (53.7%) received systemic therapy without oncologic surgery, 12 (17.9%) were prescribed chemotherapy but did not receive it, and 14 (20.9%) were not prescribed systemic therapy (**Table 3**). Fifteen patients underwent an initial surgical procedure; five were oncologic resections and 10 were non-oncologic pleurectomy/decortication procedures. Overall, 40 patients (59.7%) received chemotherapy and 11 (16.4%) received radiotherapy. Palliative care as a supportive-care service was recorded for 45 patients (67.2%) and is reported separately from anticancer treatment.

**Table 3:** Mutually exclusive anticancer treatment pathways.

Treatment pathway	n (%)
Oncologic surgery with or without systemic therapy/radiotherapy	5 (7.5)
Systemic therapy without oncologic surgery	36 (53.7)
Chemotherapy prescribed but not received	12 (17.9)
No systemic therapy prescribed	14 (20.9)

Categories sum to 67. Palliative care was a supportive-care service and was recorded separately (45/67, 67.2%). Initial surgery was recorded in 15 patients, but only five underwent oncologic resection; 10 underwent non-oncologic pleurectomy/decortication.

### Overall survival and event-free survival

All 67 patients contributed to the OS and diagnosis-based EFS analyses, and all experienced an event; there were 67 deaths and no censored OS observations. For EFS, 33 patients had documented progression before death and 34 died without a previously documented progression; there were no censored EFS observations. Reverse Kaplan–Meier median follow-up was not estimable because no observations were censored. Median OS was 9.20 months (95% CI, 6.11–14.13); OS was 43.3% (95% CI, 32.9%–56.9%) at 12 months and 13.4% (95% CI, 7.3%–24.7%) at 24 months. Median EFS was 6.51 months (95% CI, 5.49–9.13); EFS was 29.9% (95% CI, 20.7%–43.1%) at 12 months and 7.5% (95% CI, 3.2%–17.3%) at 24 months.

**Figure 2** presents unadjusted Kaplan–Meier estimates. OS did not differ clearly by PD-L1 TPS ≥1% versus <1% (two-sided log-rank p = 0.397) or by BAP1 loss versus retained among evaluable patients (p = 0.300). The corresponding EFS comparisons were p = 0.209 for PD-L1 TPS and p = 0.230 for BAP1. These unadjusted comparisons are distinct from the adjusted Cox models reported below.

**Figure 2:**
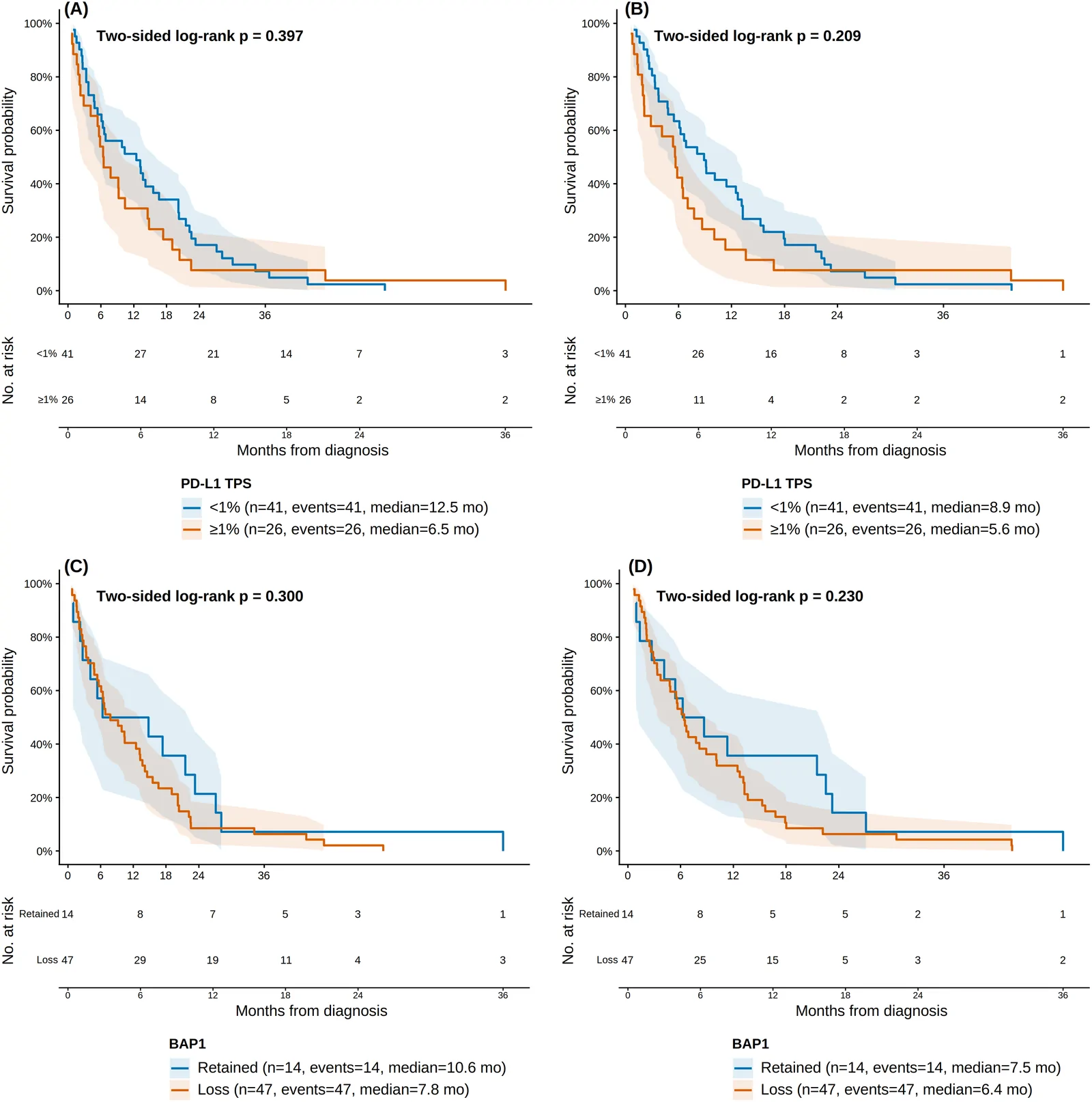
Overall survival and diagnosis-based event-free survival by PD-L1 TPS and BAP1 status. Kaplan–Meier estimates of overall survival (OS) and event-free survival (EFS). **(A)** OS by PD-L1 tumor proportion score (TPS) <1% versus ≥1% (N = 67). **(B)** EFS by PD-L1 TPS <1% versus ≥1% (N = 67). **(C)** OS by BAP1 retained versus loss among evaluable patients (N = 61). **(D)** EFS by BAP1 status among evaluable patients (N = 61). Shaded bands indicate 95% confidence intervals; tables show numbers at risk. Two-sided log-rank p values are displayed. OS was measured from pathologic diagnosis to death. EFS was measured from pathologic diagnosis to first documented progression or death. These are unadjusted associations.

Multivariable Cox results are shown in **Table 4**; **Figure 3**. In the PD-L1 models (n = 64; 64 events), PD-L1 TPS ≥1% was not clearly associated with OS (adjusted HR, 1.03; 95% CI, 0.57–1.86; p = 0.918) or EFS (adjusted HR, 1.11; 95% CI, 0.62–2.00; p = 0.717). In separate BAP1 models (n = 58; 58 events), BAP1 loss was not clearly associated with OS (adjusted HR, 1.57; 95% CI, 0.78–3.16; p = 0.205) or EFS (adjusted HR, 1.65; 95% CI, 0.82–3.33; p = 0.160). ECOG performance status 2–3 was associated with higher hazards in all four models; in the PD-L1 models, the adjusted HR was 4.53 (95% CI, 2.19–9.36; p < 0.001) for OS and 4.19 (95% CI, 2.02–8.70; p < 0.001) for EFS. Stage IV was not clearly associated with either endpoint after adjustment. The global Schoenfeld tests did not indicate violation of the proportional hazards assumption in any of the four primary models (global p values, 0.094–0.361).

**Table 4:** Adjusted Cox proportional hazards models for OS and diagnosis-based EFS.

Model/endpoint	Covariate and comparison	Adjusted HR (95% CI)	p value
PD-L1/OS	Age, per 10 years	0.95 (0.68–1.31)	0.734
	Male vs female	0.56 (0.27–1.14)	0.110
	ECOG 2–3 vs 0–1	4.53 (2.19–9.36)	<0.001
	Non-epithelioid vs epithelioid	1.51 (0.70–3.26)	0.295
	Stage IV vs I–III	1.03 (0.61–1.76)	0.910
	PD-L1 TPS ≥1% vs <1%	1.03 (0.57–1.86)	0.918
PD-L1/EFS	Age, per 10 years	0.96 (0.69–1.33)	0.809
	Male vs female	0.69 (0.32–1.49)	0.345
	ECOG 2–3 vs 0–1	4.19 (2.02–8.70)	<0.001
	Non-epithelioid vs epithelioid	1.83 (0.77–4.36)	0.175
	Stage IV vs I–III	0.95 (0.57–1.61)	0.855
	PD-L1 TPS ≥1% vs <1%	1.11 (0.62–2.00)	0.717
BAP1/OS	Age, per 10 years	0.96 (0.68–1.36)	0.826
	Male vs female	0.74 (0.34–1.61)	0.449
	ECOG 2–3 vs 0–1	4.91 (2.29–10.54)	<0.001
	Non-epithelioid vs epithelioid	2.03 (0.82–5.01)	0.124
	Stage IV vs I–III	1.08 (0.61–1.92)	0.790
	BAP1 loss vs retained	1.57 (0.78–3.16)	0.205
BAP1/EFS	Age, per 10 years	0.98 (0.70–1.37)	0.901
	Male vs female	0.84 (0.37–1.91)	0.681
	ECOG 2–3 vs 0–1	4.70 (2.18–10.11)	<0.001
	Non-epithelioid vs epithelioid	1.92 (0.74–5.00)	0.182
	Stage IV vs I–III	0.96 (0.55–1.67)	0.873
	BAP1 loss vs retained	1.65 (0.82–3.33)	0.160

PD-L1 models included 64 patients and 64 events; three patients with missing stage were excluded. BAP1 models included 58 patients and 58 events; six patients without evaluable BAP1 and three with missing stage were excluded. Each model contained six parameters. Treatment and platelet count were omitted. HR, hazard ratio; CI, confidence interval; OS, overall survival; EFS, event-free survival; ECOG, Eastern Cooperative Oncology Group; TPS, tumor proportion score.

**Figure 3:**
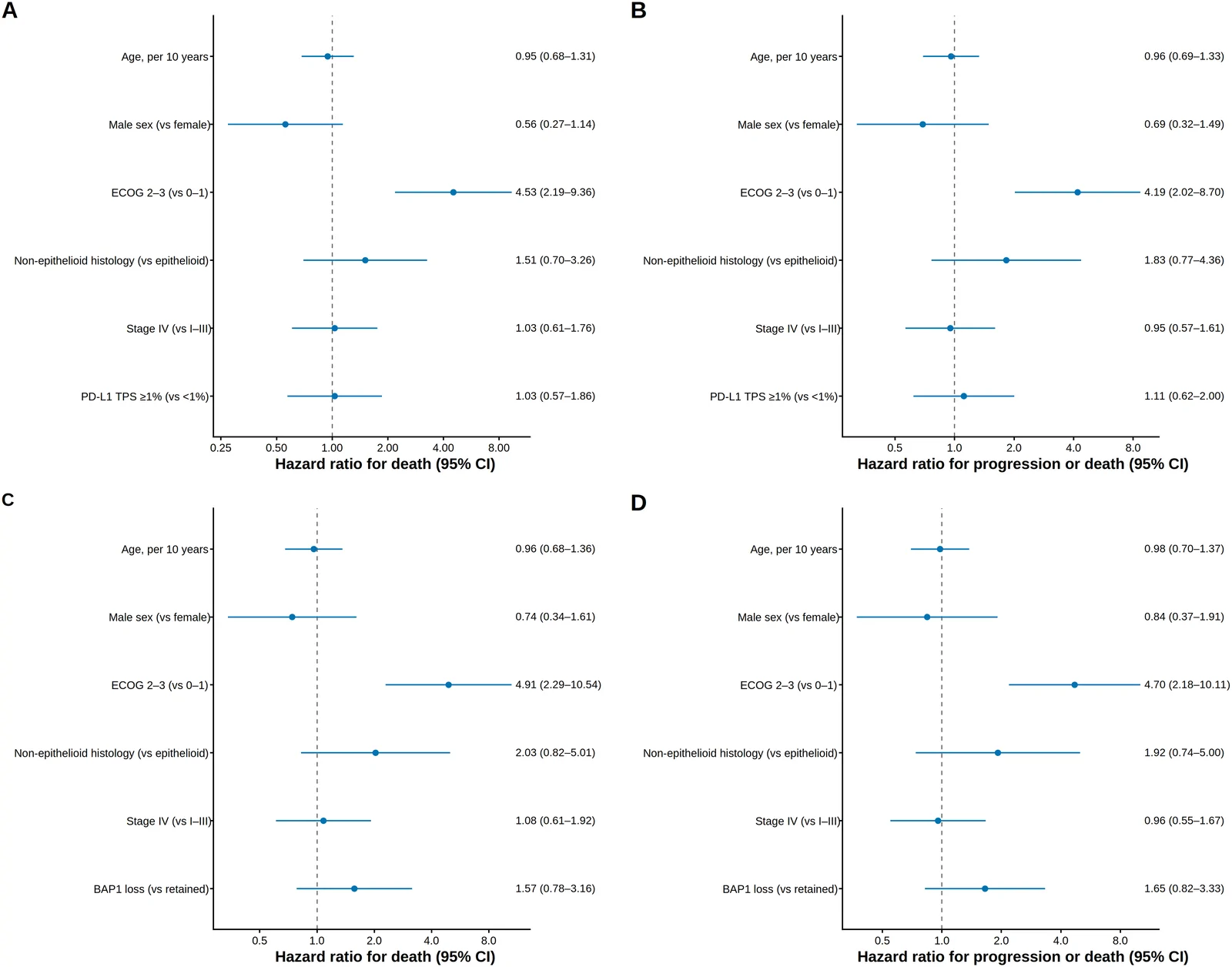
Adjusted Cox models for overall survival and diagnosis-based event-free survival. Forest plots show hazard ratios (HRs) and 95% confidence intervals. **(A)** Overall survival (OS) model for PD-L1 TPS ≥1% versus <1% (n = 64; 64 events). **(B)** Event-free survival (EFS) model for PD-L1 TPS ≥1% versus <1% (n = 64; 64 events). **(C)** OS model for BAP1 loss versus retained (n = 58; 58 events). **(D)** EFS model for BAP1 loss versus retained (n = 58; 58 events). All models included age per 10 years, sex, ECOG 2–3 versus 0–1, non-epithelioid versus epithelioid histology, and stage IV versus I–III. Missing stage was excluded. Treatment and platelet count were not included. HRs represent adjusted associations, not causal effects.

Exploratory combined-biomarker and histology-stratified Kaplan–Meier analyses are presented in **Supplementary Figures S1, S2**; small group sizes precluded adjusted or interaction analyses.

### Exploratory PD-L1 TPS thresholds of ≥5% and ≥10%

Exploratory analyses at PD-L1 TPS ≥5% (n = 14) and ≥10% (n = 7) are reported in **Supplementary Table S3**; **Supplementary Figure S3**. In unadjusted Cox analyses, TPS ≥5% had HRs of 1.93 (95% CI, 1.04–3.60; p = 0.038) for OS and 2.13 (95% CI, 1.14–3.96; p = 0.018) for EFS. TPS ≥10% had HRs of 2.13 (95% CI, 0.95–4.74; p = 0.065) for OS and 2.30 (95% CI, 1.02–5.18; p = 0.044) for EFS. Because these analyses used sparse groups and multiple thresholds, they were not interpreted as a dose-response and adjusted models were not fitted for these thresholds because of the small exposed groups.

## Discussion

In this retrospective Colombian cohort, PD-L1 TPS ≥1% was present in 38.8% of tumors and BAP1 loss in 77.0% of evaluable tumors. Neither biomarker showed a clear adjusted association with OS or diagnosis-based EFS. ECOG 2–3 was associated with higher hazards in the multivariable models, whereas the stage estimates were close to the null after adjustment. The higher PD-L1 thresholds produced larger unadjusted estimates, but those analyses involved only 14 and 7 patients and were exploratory. The combined PD-L1/BAP1 analysis also did not provide evidence of added prognostic information.

The prevalence of PD-L1 TPS ≥1% was broadly consistent with estimates of approximately 40%–52% in recent cohorts (6, 7). A recent IASLC staging-project cohort reported PD-L1 expression in 89 of 200 tumors (44.5%) and BAP1 loss by immunohistochemistry in 260 of 452 tumors (57.5%), providing a larger international comparator and further illustrating cross-cohort heterogeneity (20). Comparisons across studies remain limited by differences in antibody clone, scoring, threshold, histologic composition, and covariate adjustment.

Previous quantitative syntheses have generally suggested an adverse prognostic association for PD-L1 expression. Zhang and Gong pooled 11 studies comprising 1,606 patients and found that elevated PD-L1 expression was associated with poorer OS (HR 1.50, 95% CI 1.32–1.70; p<0.001) (21). Similarly, Jin et al. reported an adverse association between elevated PD-L1 expression and OS in a meta-analysis of 16 studies (10). Importantly, PD-L1 expression has also been associated with non-epithelioid histology, making histologic composition a potentially important source of confounding. Brcic et al. reported shorter survival with expression above 10% (12). Conversely, a systematic review concluded that the available evidence was not sufficiently consistent to establish a definitive prognostic or predictive role (11). More recently, the large IASLC staging-project cohort found no significant association between PD-L1 expression and OS (20), further illustrating the heterogeneity of the available evidence. Thus, although previous pooled evidence supports an adverse prognostic association, more recent data continue to show uncertainty regarding the independent prognostic value of PD-L1. Our adjusted estimates at the primary 1% threshold support that cautious interpretation rather than a claim of established prognostic value.

The exploratory ≥5% and ≥10% findings should be read separately from the primary analysis. Alternative thresholds have been used in prior studies, including 10% and 15% (12, 16), but no single cutoff is uniformly established for prognostic assessment in PM. In this cohort, only 14 patients had TPS ≥5% and seven had TPS ≥10%. Testing several thresholds in a small sample increases the probability of unstable or chance findings. The observed sequence of HRs therefore does not demonstrate a dose-response relationship.

Studies of germline BAP1 alterations have established the broader biological relevance of BAP1 (22, 23), whereas clinicopathologic studies have associated immunohistochemical BAP1 loss with longer survival or improved outcomes after first-line platinum–pemetrexed chemotherapy (8, 9). Other cohorts have not identified a clear independent prognostic association (6, 7). The wide confidence intervals in our BAP1 models allow for both lower and higher hazards, so absence of a conventional statistical association should not be interpreted as proof of no association.

PD-L1 and BAP1 were evaluated together because they represent distinct biological domains (immune evasion and tumor-suppressor loss) and could, in principle, provide complementary information. Both markers were evaluable in 61 patients, but two of the four combined groups contained only seven patients. We therefore limited the analysis to exploratory Kaplan–Meier descriptions and did not fit a four-group adjusted Cox model. The absence of added information is consistent with a recent small cohort that assessed the same marker pair (7), but the available studies remain too small to exclude clinically relevant joint associations.

Median OS was 9.20 months, compared with 16.2 months (95% CI, 13.8–18.5) in the Mexican PLECH cohort of patients receiving first-line chemotherapy (17). These populations are not directly comparable: the present cohort included all treatment pathways, whereas PLECH required first-line chemotherapy. Differences in eligibility, stage, performance status, calendar period, and treatment access may contribute to the survival difference; the available data do not support attribution to any single factor. Our treatment description therefore remains descriptive and treatment was excluded from the primary prognostic models because of confounding by indication.

Asbestos exposure was documented in 32/67 patients through clinical-record review. Colombia prohibited the exploitation, production, commercialization, import, and export of asbestos under Law 1968 of 2019, effective January 1, 2021 (24). This change occurred near the end of the 2011–2023 study period and, given the long latency of PM, does not explain the recorded exposure frequency. The more cautious interpretation is under-ascertainment in retrospective clinical histories, particularly when occupational detail was incomplete.

The study evaluates prognostic association, not prediction of benefit from immune checkpoint inhibitors. CheckMate 743 established nivolumab plus ipilimumab as a first-line option for unresectable PM, but PD-L1 has not been validated as a stand-alone treatment-selection biomarker (13, 14). These data should therefore not be used to recommend or withhold immunotherapy on the basis of PD-L1 or BAP1 status. The contribution of this cohort is narrower: it provides jointly measured biomarker frequencies, transparent denominators, and adjusted estimates from a Colombian referral population while showing that the apparent PD-L1 association is sensitive to threshold and model specification.

### Limitations

This study has several limitations. Its retrospective single-center design and referral setting limit generalizability and may introduce selection bias. The screening log and consecutive ascertainment were unavailable, and tissue availability restricted BAP1 analyses to 61 patients. The cohort and non-epithelioid subgroups were small; complete-case BAP1 models had fewer than 10 events per parameter, leaving wide confidence intervals and possible model instability. Treatments and specimen sources were heterogeneous across a 13-year period in which treatment standards changed. Treatment allocation was vulnerable to confounding by indication, and residual confounding remains likely despite adjustment. Asbestos exposure was obtained from clinical records and may be under-recorded. Multiple exploratory PD-L1 thresholds and small combined-biomarker groups increase the risk of chance findings; no external cohort was available for validation. Finally, all patients had died, so reverse Kaplan–Meier median follow-up was not estimable. EFS included death when progression had not previously been documented in 34 patients, which may partly reflect incomplete progression ascertainment rather than observed progression.

## Conclusions

In this retrospective Colombian cohort, neither PD-L1 TPS ≥1% nor BAP1 loss showed a clear adjusted association with OS or diagnosis-based EFS. Results at the exploratory PD-L1 thresholds of ≥5% and ≥10% were based on small groups and do not establish a dose-response. The combined biomarkers did not provide additional prognostic information. These findings require external validation in larger, multicenter cohorts.

## Data Availability

The raw data supporting the conclusions of this article will be made available by the authors, without undue reservation.
