# Supplementary material for: Prognostic associations of PD-L1 expression and BAP1 loss in pleural mesothelioma: a retrospective single-center cohort from Colombia
Source: Front Oncol. 2026 Oct 14;16:1920798. doi: 10.3389/fonc.2026.1920798 (PMC13713424; doi:10.3389/fonc.2026.1920798)
Supplement: Supplementary file 1 [file DataSheet1.docx]

Supplementary material

**Supplementary Table S1. Baseline characteristics by PD-L1 TPS ≥1%.**

| **Variable** | **Category/statistic** | **TPS <1%** | **TPS ≥1%** | **p value** |
| --- | --- | --- | --- | --- |
| N |  | 41 | 26 | — |
| Age, years | Median (IQR) | 68.0 (60.0–74.0) | 62.5 (56.2–69.5) | 0.070 |
| Sex | Female | 11/41 (26.8) | 4/26 (15.4) | 0.372 |
|  | Male | 30/41 (73.2) | 22/26 (84.6) | — |
| ECOG | 0–1 | 31/41 (75.6) | 19/26 (73.1) | >0.9 |
|  | 2–3 | 10/41 (24.4) | 7/26 (26.9) | — |
| Histology | Epithelioid | 36/41 (87.8) | 21/26 (80.8) | 0.493 |
|  | Non-epithelioid | 5/41 (12.2) | 5/26 (19.2) | — |
| Grouped stage | Early | 3/41 (7.3) | 1/26 (3.8) | 0.360 |
|  | Locally advanced | 11/41 (26.8) | 11/26 (42.3) | — |
|  | Metastatic | 26/41 (63.4) | 12/26 (46.2) | — |
|  | Missing | 1/41 (2.4) | 2/26 (7.7) | — |
| Exposure history | Asbestos only | 6/41 (14.6) | 4/26 (15.4) | 0.904 |
|  | Tobacco only | 10/41 (24.4) | 5/26 (19.2) | — |
|  | Asbestos and tobacco | 12/41 (29.3) | 10/26 (38.5) | — |
|  | Neither documented | 13/41 (31.7) | 7/26 (26.9) | — |

*Percentages use the column denominator. p values are two-sided; one p value is reported per variable. IQR, interquartile range; ECOG, Eastern Cooperative Oncology Group; TPS, tumor proportion score.*

Supplementary Table S2. Baseline characteristics by BAP1 status among evaluable tumors.

| **Variable** | **Category/statistic** | **BAP1 retained** | **BAP1 loss** | **p value** |
| --- | --- | --- | --- | --- |
| N |  | 14 | 47 | — |
| Age, years | Median (IQR) | 67.0 (58.0–74.2) | 66.0 (58.0–72.0) | >0.9 |
| Sex | Female | 1/14 (7.1) | 13/47 (27.7) | 0.156 |
|  | Male | 13/14 (92.9) | 34/47 (72.3) | — |
| ECOG | 0–1 | 11/14 (78.6) | 35/47 (74.5) | >0.9 |
|  | 2–3 | 3/14 (21.4) | 12/47 (25.5) | — |
| Histology | Epithelioid | 13/14 (92.9) | 39/47 (83.0) | 0.670 |
|  | Non-epithelioid | 1/14 (7.1) | 8/47 (17.0) | — |
| Grouped stage | Early | 1/14 (7.1) | 3/47 (6.4) | 0.263 |
|  | Locally advanced | 3/14 (21.4) | 16/47 (34.0) | — |
|  | Metastatic | 8/14 (57.1) | 27/47 (57.4) | — |
|  | Missing | 2/14 (14.3) | 1/47 (2.1) | — |
| Exposure history | Asbestos only | 2/14 (14.3) | 8/47 (17.0) | 0.330 |
|  | Tobacco only | 2/14 (14.3) | 13/47 (27.7) | — |
|  | Asbestos and tobacco | 7/14 (50.0) | 11/47 (23.4) | — |
|  | Neither documented | 3/14 (21.4) | 15/47 (31.9) | — |

*The analysis includes 61 patients with evaluable BAP1. Percentages use the column denominator. IQR, interquartile range; ECOG, Eastern Cooperative Oncology Group.*

Supplementary Table S3. Exploratory unadjusted Cox analyses at higher PD-L1 TPS thresholds.

| **Threshold** | **Endpoint** | **Positive n/N** | **Unadjusted HR (95% CI)** | **p value** |
| --- | --- | --- | --- | --- |
| TPS ≥5% | OS | 14/67 | 1.93 (1.04–3.60) | 0.038 |
| TPS ≥5% | EFS | 14/67 | 2.13 (1.14–3.96) | 0.018 |
| TPS ≥10% | OS | 7/67 | 2.13 (0.95–4.74) | 0.065 |
| TPS ≥10% | EFS | 7/67 | 2.30 (1.02–5.18) | 0.044 |

*These analyses are exploratory. Only 14 and 7 patients met the ≥5% and ≥10% thresholds, respectively. Multiple thresholds were tested; estimates must not be interpreted as a dose-response. Adjusted threshold models are omitted.*

Supplementary Table S4. Combined PD-L1/BAP1 exploratory groups.

| **Group** | **n** | **OS events** | **EFS events** |
| --- | --- | --- | --- |
| PD-L1 TPS <1% and BAP1 loss | 30 | 30 | 30 |
| PD-L1 TPS <1% and BAP1 retained | 7 | 7 | 7 |
| PD-L1 TPS ≥1% and BAP1 loss | 17 | 17 | 17 |
| PD-L1 TPS ≥1% and BAP1 retained | 7 | 7 | 7 |

*Both biomarkers were evaluable in 61 patients. No four-group adjusted Cox model was fitted because two groups contained only seven patients.*

Supplementary Figure S1. Exploratory Kaplan–Meier curves by combined PD-L1 TPS and BAP1 status.

*(A) Overall survival. (B) Diagnosis-based event-free survival. Analyses include the 61 patients with both biomarkers evaluable. Group sizes are reported in Supplementary Table S4 and on the figure. No adjusted four-group model was fitted because of the small group sizes; these curves do not establish independent or predictive biomarker information.*

Supplementary Figure S2. Exploratory overall survival by histologic subtype.

*The non-epithelioid group included 10 patients. This unadjusted analysis is descriptive and does not test biomarker–histology interaction.*

Supplementary Figure S3. Exploratory Kaplan–Meier curves at PD-L1 TPS thresholds of ≥5% and ≥10%. (A) OS for TPS ≥5% versus <5%.

*(B) EFS for TPS ≥5% versus <5%. (C) OS for TPS ≥10% versus <10%. (D) EFS for TPS ≥10% versus <10%. Only 14 and 7 patients met the ≥5% and ≥10% thresholds, respectively. Two-sided log-rank p values are shown. These analyses are exploratory and do not demonstrate a dose-response.*
